# Activated circulating T follicular helper 17 cells positively correlated with anti-HBV humoral immunity in chronic hepatitis B patients

**DOI:** 10.3389/fmicb.2025.1708034

**Published:** 2026-01-13

**Authors:** Qi Gu, Minxin Mao, Yuan Liu, Xin Tong, Juan Zhang, Jinqiu Ran, Xiaoyan Ma, Juan Xia, Rui Huang, Jie Li, Tianyang Liu, Yuxin Chen, Shengxia Yin, Chao Wu

**Affiliations:** 1 Department of Infectious Diseases, Nanjing Drum Tower Hospital, Affiliated Hospital of Medical School, Nanjing University, Nanjing, China; 2 Department of Infectious Diseases, Nanjing Drum Tower Hospital, Affiliated Hospital of Nanjing University of Chinese Medicine, Nanjing, Jiangsu, China; 3 Department of Laboratory Medicine, Nanjing Drum Tower Hospital, Affiliated Hospital of Medical School, Nanjing University, Nanjing, Jiangsu, China; 4 Institute of Viruses and Infectious Diseases, Nanjing University, Nanjing, Jiangsu, China; 5 Department of Laboratory Medicine, Joint Institute of Nanjing Drum Tower Hospital for Life and Health, College of Life Science, Nanjing Normal University, Nanjing, Jiangsu, China

**Keywords:** follicular helper T 17 cells, follicular helper T cells, hepatitis B virus, humoral immunity, interleukin-21

## Abstract

**Introduction:**

Dysfunction of hepatitis B virus (HBV)-specific B cells and lack of antibodies against hepatitis B surface antigen (HBsAg) are associated with failure to achieve functional cure in chronic hepatitis B (CHB). Follicular helper T (Tfh) cells are essential for B-cell differentiation into plasma cells and comprise three subsets: Tfh1, Tfh2, and Tfh17 cells. Our previous studies suggested dysregulated Tfh responses in CHB patients. However, the functions of Tfh cell subsets in CHB progression and treatment remain incompletely characterized.

**Methods:**

To explore the role of Tfh subgroups in HBV infection, we analyzed the frequencies of total and HBsAg-specific Tfh cell subsets and their surface markers using flow cytometry.

**Results:**

Compared with healthy individuals [healthy controls (HCs)], CHB patients had significantly higher frequencies of total Tfh cells, lower frequencies of Tfh17 cells and increased PD-1 expression. The frequency of quiescent Tfh17 cells negatively correlated with HBsAg^+^ B cells and positively correlated with total immunoglobulin G (IgG). Further analysis revealed positive correlations between the frequency of quiescent Tfh17 cells and IgG1 and IgG3 levels.

**Discussion:**

These results suggest that Tfh17 frequency varies across immune phases in chronic HBV infection and that Tfh17 cells correlate with the immune response against chronic HBV infection.

**Importance:**

HBV is a major global public health problem. Dysfunction of HBV-specific B cells is an important reason for the failure to achieve a functional cure for HBV. Tfh cells are dysregulated in CHB patients, potentially leading to impaired B-cell differentiation into plasma cells. Here, we found that although Tfh17 cell frequencies were decreased in CHB patients, they exhibited a more activated state. Activated Tfh17 cells correlated positively with HBsAg levels, while quiescent Tfh17 cells correlated negatively. Furthermore, CHB patients had higher frequencies of HBV-specific Tfh17 cells, which positively correlated with serum HBsAg and HBV DNA levels. Our study provides new insights into the role of Tfh17 cells in CHB infection, potentially informing immunotherapeutic strategies for functional cure.

## Introduction

Hepatitis B virus (HBV) is a major global public health problem, chronically infecting over 257.5 million people worldwide (Polaris Observatory Collaborators, 2023). In 2022, an estimated 254 million people were living with chronic hepatitis B, resulting in approximately 1.1 million deaths due to HBV-associated cirrhosis and hepatocellular carcinoma (HCC) (Easterbrook et al., 2024; Burki, 2024). The clinical outcome of HBV infection depends on the interplay between viral replication and the host immune response. While the phenotype and function of virus-specific CD4^+^ and CD8^+^ T cells in chronic hepatitis B (CHB) have been extensively studied, recent research also highlights virus-specific B-cell dysfunction (Li et al., 2020; Liu et al., 2019; Tian et al., 2018; Burton et al., 2018; Salimzadeh et al., 2018).

T follicular helper (Tfh) cells, a subset of T cells, support B-cell differentiation in germinal centers (GCs) and play a crucial role in the seroconversion of hepatitis B e antigen (HBeAg) and hepatitis B surface antigen (HBsAg) in CHB patients (Wang et al., 2018; Wang et al., 2021; Vyas et al., 2021). Tfh cells express high levels of chemokine receptor 5 protein (CXCR5) and the transcription factor B-cell lymphoma 6 (BCL6). Other important markers include C-X-C chemokine receptor type 3 (CXCR3), inducible T-cell costimulator (ICOS), programmed cell death-1 (PD-1) (Crotty, 2011; Jogdand et al., 2016). Circulating Tfh (cTfh) cells can exhibit activated or quiescent phenotypes based on expression levels of PD-1, ICOS, or C-C motif chemokine receptor 7 (CCR7) (Hale and Ahmed, 2015; Schmitt et al., 2014). Based on CXCR3 and CCR6 expression, Tfh cells are further subdivided into Tfh1 (CXCR3^+^CCR6^−^), Tfh2 (CXCR3^−^CCR6^−^), and Tfh17 (CXCR3^−^CCR6^+^). The subgroups of Tfh cells have different functions: Tfh1 cells express the transcription factor T-bet and produce the cytokine IFN-*γ*; Tfh2 cells express the transcription factor GATA3 and produce the cytokines IL-4, IL-5, and IL-13; Tfh17 cells express the transcription factor RORγT and produce the cytokines IL-17A and IL-22 (Kurata et al., 2021). However, their specific roles in supporting HBV-specific B cells and modulating chronic HBV infection remain unclear.

PD-1 and ICOS are crucial molecules regulating Tfh cells, with their expression levels directly influencing Tfh cell function and activity. When PD-1 acts as a ligand for co-inhibitory receptors on antigen-presenting cells (Yokosuka et al., 2012; Zinselmeyer et al., 2013), it forms a TCR-PD-1 signaling cluster in Tfh cells. This complex recruits SHP2 to reduce downstream phosphorylation in the TCR signaling pathway, thereby blocking nuclear factor-kappa B (NF-Κb) signaling. PD-1 suppresses Tfh cell development through this pathway, and blocking PD-1 can increase Tfh cell proliferation by 2–3 times in proportion and 6–10 times in quantity. However, PD-1 also plays a vital role in promoting Tfh cell function, as its presence is essential for Tfh cells to secrete IL-21. ICOS serves as a key marker for Tfh generation and germinal center (GC) formation. ICOS downregulates Krüppel-like factor 2 (Klf2) via Foxo1, which negatively regulates CXCR5. When ICOS signaling is inhibited, Klf2 expression increases, leading to rapid upregulation of T cell homing factors such as CCR7 and P-selectin glycoprotein ligand-1 (PSGL-1), while the number of Tfh cells and antigen-specific GC B cells decreases sharply (Weber et al., 2015). Additionally, ICOS induces rapid exocytosis of CD40L from intracellular storage. Under the influence of CD40L, GC B cells can upregulate ICOS ligands (ICOSL), thereby maintaining contact between GC Tfh cells and B cells (Liu et al., 2015). ICOS also serves as a key biomarker for Tfh cell function. Studies have demonstrated that ICOS can induce upregulation of the transcription factor c-Maf and IL-21 expression in both Tfh and Th17 cells (Bauquet et al., 2009). The PI3K signaling pathway is another mechanism through which ICOS regulates IL-21 secretion. ICOS contains a PI3K-binding domain that, upon binding to PI3K, triggers CD28 activation and subsequently promotes IL-21 secretion (Gigoux et al., 2009).

Different Tfh subsets exhibit distinct functionalities in various chronic infections, including coronavirus disease 2019 (COVID-19), hepatitis C virus (HCV), and human immunodeficiency virus (HIV). Acute COVID-19 is associated with decreased Tfh1 and increased Tfh17 cells (Golovkin et al., 2021), with majority of the SARS-CoV-2-specific Tfh cells belonging to the Tfh17 subset (Juno et al., 2020). Conversely, in HIV patients, the Tfh cells expanded, and more HIV-specific Tfh cells had a Tfh1 polarization with a high expression of Tfh-related functions (Niessl et al., 2020). In HCV infection, Tfh cells differentiate towards CXCR3^+^ Tfh cells, which positively correlate with the HCV antibody response (Zhang et al., 2019). However, the specific roles of Tfh subsets in HBV immune responses are less defined. Given the importance of Tfh cells in HBV immune regulation, characterizing their phenotypes and functions, particularly Tfh17 cells, is necessary.

In this study, we evaluated the frequency and phenotype of Tfh cell subsets, especially Tfh17 cells, in CHB patients. Our results indicate that Tfh17 frequency varies across immune stages in chronic HBV infection and contributes to the anti-HBV immune response.

## Results

### Clinical characteristics of CHB patients in different immune phases

Clinical characteristics of CHB patients and healthy controls (HCs) are summarized in Table 1. In the 68 CHB patients, 3 were in the immune-tolerant (IT) phase, 16 in the HBeAg-positive immune-active (EPH) phase, 11 in the HBeAg-negative immune reactivation (ENH) phase, and 38 in the inactive HBV carrier (IC) phase. HC were HBsAg-negative with normal ALT levels. ALT and AST levels were significantly higher in the EPH phase than in the IT, IC, and HC groups (Table 1). Overall, 46 (67.6%) patients were male, and 27 (39.7%) patients were HBeAg-positive. The median ages were 33, 34, 47, and 52 years for IT, EPH, IC, and ENH patients, respectively. The median age was 36 years for HC. The median serum HBV DNA (7.55 log10 IU/ml) and HBsAg (48076.76 IU/mL) were highest in the IT phase.

**Table 1:** Clinical characteristics of patients with CHB infection in different immune statuses.

Characteristic	IT (*n* = 3)	EPH (*n* = 16)	IC (*n* = 38)	ENH (*n* = 11)	HC (*n* = 20)
Male (*n*, %)	2 (66.67%)	12 (75.00%)	23 (60.53%)	9 (81.82%)	7 (35.00%)
Age (years)	33.00 (29.00–37.00)	34.00 (31.00–46.00)	47.00 (36.25–56.50)	52.00 (41.00–53.00)	36.00 (30.50–42.00)
HBsAg (IU/mL)	48076.76 (31028.17–79372.99)	21812.62 (3324.10–51367.59)	538.40 (35.58–2157.04)	2014.83 (1097.07–2902.14)	(—)
ALT (U/L)	31.40 (25.07–39.20)	77.00 (53.20–169.10)	23.35 (17.40–37.57)	65.90 (46.80–68.80)	13.80 (10.37–18.25)
AST (U/L)	21.95 (20.07–29.00)	56.30 (31.20–98.70)	23.25 (20.30–28.15)	37.50 (29.70–48.60)	15.90 (13.42–19.45)
HBV DNA (log_10_IU/mL)	7.55 (7.37–7.85)	7.21 (5.68–8.07)	2.70 (2.69–2.81)	4.39 (3.23–5.66)	(—)

Values were shown as *n* (%) or medians with interquartile ranges (IQRs). IT, immune-tolerant phase; EPH, HBeAg-positive immune-active phase; IC, inactive HBV carrier phase; ENH, HBeAg-negative immune reactivation phase; HCs, healthy controls; ALT, alanine aminotransferase; AST, aspartate aminotransferase; HBV, hepatitis B virus.

### Circulating Tfh and Tfh17 cells are altered in CHB patients and exhibit high PD-1 and ICOS expression

**Figure 1:**
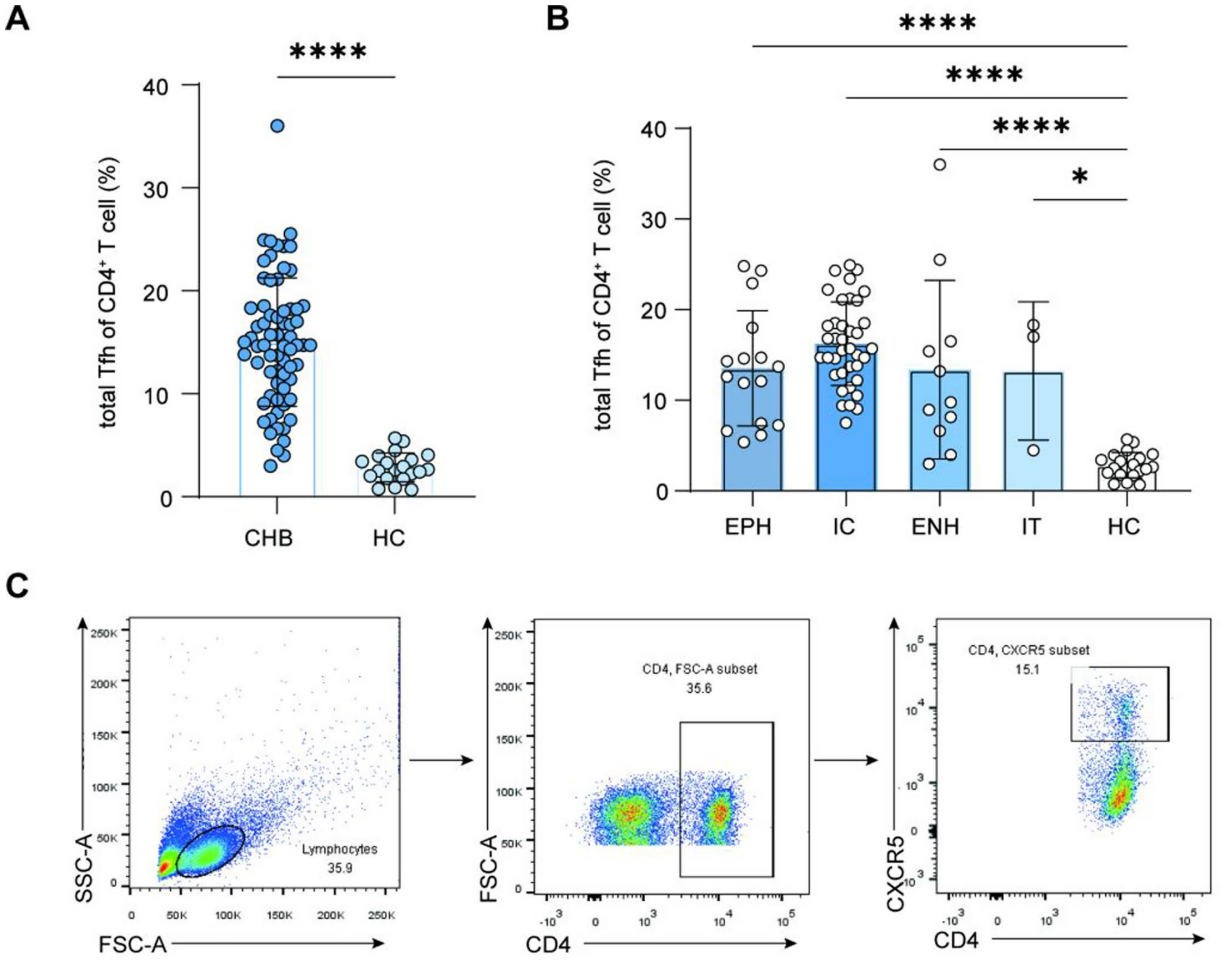
Frequency of Tfh cells in patients with chronic HBV infection. **(A)** Frequency of Tfh cells in patients with CHB and in the HC group; unpaired *t*-test, bars represent means ± standard deviation (SD). **(B)** Frequencies of Tfh cells in patients with CHB at different immunological stages and in the HC group; one-way ANOVA test, bars represent means ± SD. **(C)** Representative gating strategy for Tfh cells analysis. Tfh, follicular T helper; HBV, hepatitis B virus; CHB, chronic hepatitis B; HC, healthy control; IT, immune-tolerant phase; EPH, HBeAg-positive immune-active phase; IC, inactive CHB carrier phase; ENH, HBeAg-negative immune reactivation phase. ^*^p < 0.05, ^**^p < 0.01, ^***^p < 0.001, and ^****^p < 0.0001.

We assessed the frequency of the cTfh cells in peripheral blood from HCs and CHB patients by flow cytometry. The results showed that the frequency of cTfh cells was increased in CHB patients compared with the HC group (15.02 vs. 2.85%, *p* < 0.0001, means) (Figure 1A). In addition, the frequency of cTfh cells was predominantly increased in the EPH group, IC group, ENH group, and IT group compared with the HC group (EPH: 13.55% vs. 2.85%, *p* < 0.0001; IC: 16.25% vs. 2.85%, *p* < 0.0001; ENH: 13.37% vs. 2.85%, *p* < 0.0001; IT: 13.26% vs. 2.85%, *p* = 0.0248; means) (Figure 1B). The representative gating strategy for Tfh cells analysis was showed in (Figure 1C).

**Figure 2:**
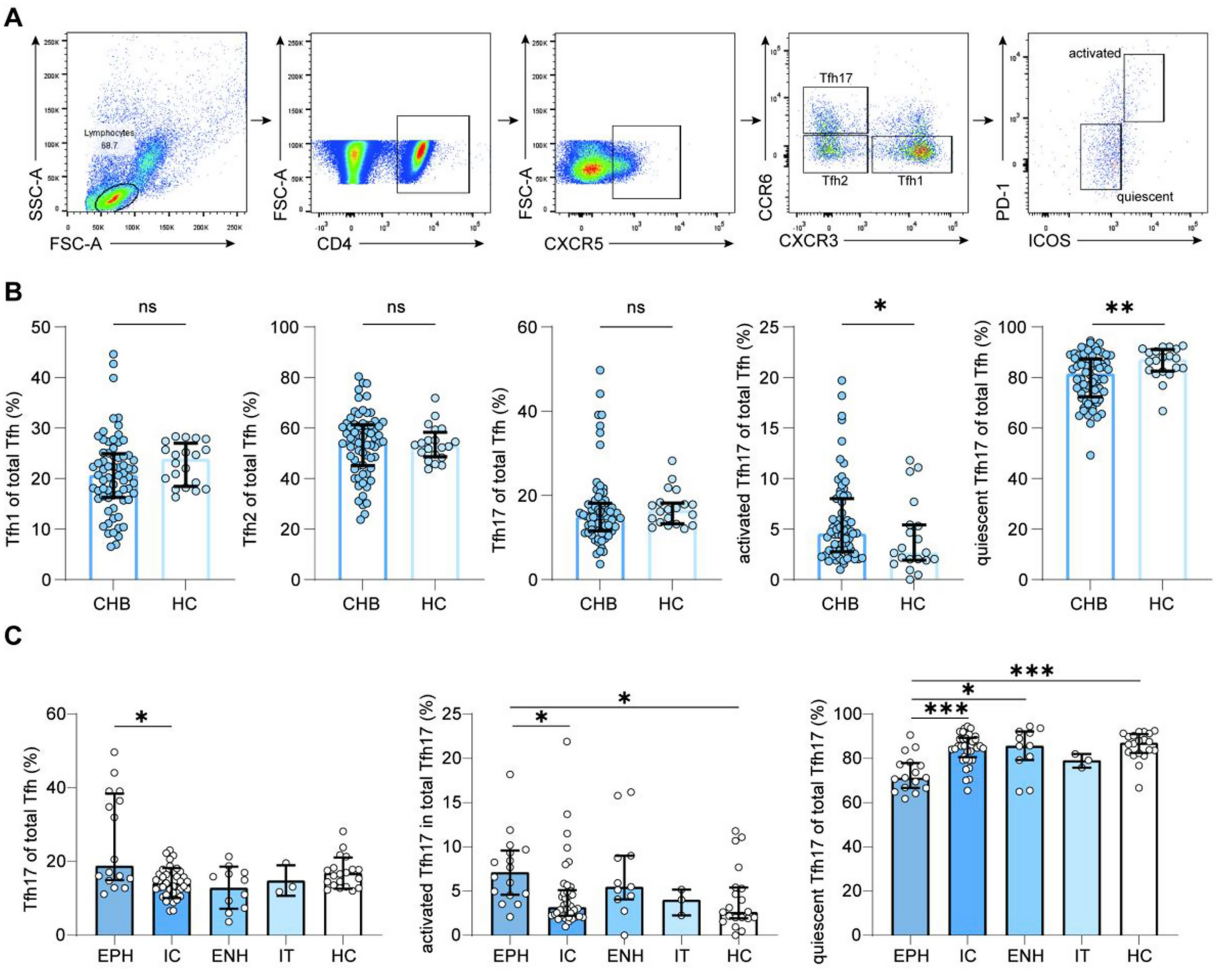
Frequency of Tfh17 and Tfh17 subset cells in patients with chronic HBV infection. **(A)** Representative gating strategy for Tfh cells and Tfh17 cells subset analysis; Mann–Whitney *U* test, bars represent medians with IQR. **(B)** Frequencies of Tfh cell subpopulations in patients with CHB. The number for each group: CHB (*n* = 68), HC (*n* = 20); Kruskal–Wallis test, bars represent medians with IQR. **(C)** Frequencies of Tfh17 and its subpopulations in patients with CHB and in the HC group at different immunological stages. The number of each group: EPH (*n* = 16), IC (*n* = 38), ENH (*n* = 11), IT (*n* = 3), HC (*n* = 20). Tfh, follicular T helper cell; HBV, hepatitis B virus; CHB, chronic hepatitis B; HC, healthy control; IT, immune-tolerant phase; EPH, HBeAg-positive immune-active phase; IC, inactive CHB carrier phase; ENH, HBeAg-negative immune reactivation phase; ^*^p < 0.05, ^**^p < 0.01, ^***^p < 0.001, and ^****^p < 0.0001.

Circulating Tfh subpopulations were identified based on CXCR3 and CCR6 expression: cTfh1 (CXCR3^+^CCR6^−^), cTfh2 (CXCR3^−^CCR6^−^), and cTfh17 (CXCR3^−^CCR6^+^) (Figure 2A). The percentage of cTfh cell subpopulations in CHB patients showed no difference in cTfh1 and cTfh2. Whereas, the frequencies of cTfh17 cells in CHB patients were lower than in HC, although there was no statistical difference (14.90% vs. 16.45%, *p* = 0.1997; medians). Further characteristics investigation showed that the frequencies of activated cTfh17 in CHB patients were significantly increased in CHB patients than in HC group (4.54% vs. 2.66%, *p* = 0.0349; medians), but the frequencies of quiescent cTfh17 cells were significantly decreased in CHB patients than in HC group (81.45% vs. 87.15%, *p* = 0.0094; medians) (Figure 2B).

We analyzed total cTfh17 and subset frequencies across immune phases. Compared to HC, the activated cTfh17 cells in EPH increased (7.14% vs. 2.66%, *p* = 0.0152; medians), and the quiescent cTfh17 cells were decreased in EPH (71.30% vs. 87.15%, *p* = 0.0005; medians). And comparing these frequencies in the four CHB immune phases, EPH patients had significantly higher frequencies of total cTfh17 and activated cTfh17 cells than IC patients (total cTfh17: 18.85% vs. 13.75%, *p* = 0.0120; activated cTfh17: 7.14% vs. 3.17%, *p* = 0.0338; medians). Quiescent cTfh17 frequencies were lower in EPH than in ENH and IC patients (ENH: 71.30% vs. 85.80%, *p* = 0.0138; IC: 71.30% vs. 85.60%, *p* = 0.0008; medians) (Figure 2C).

**Figure 3:**
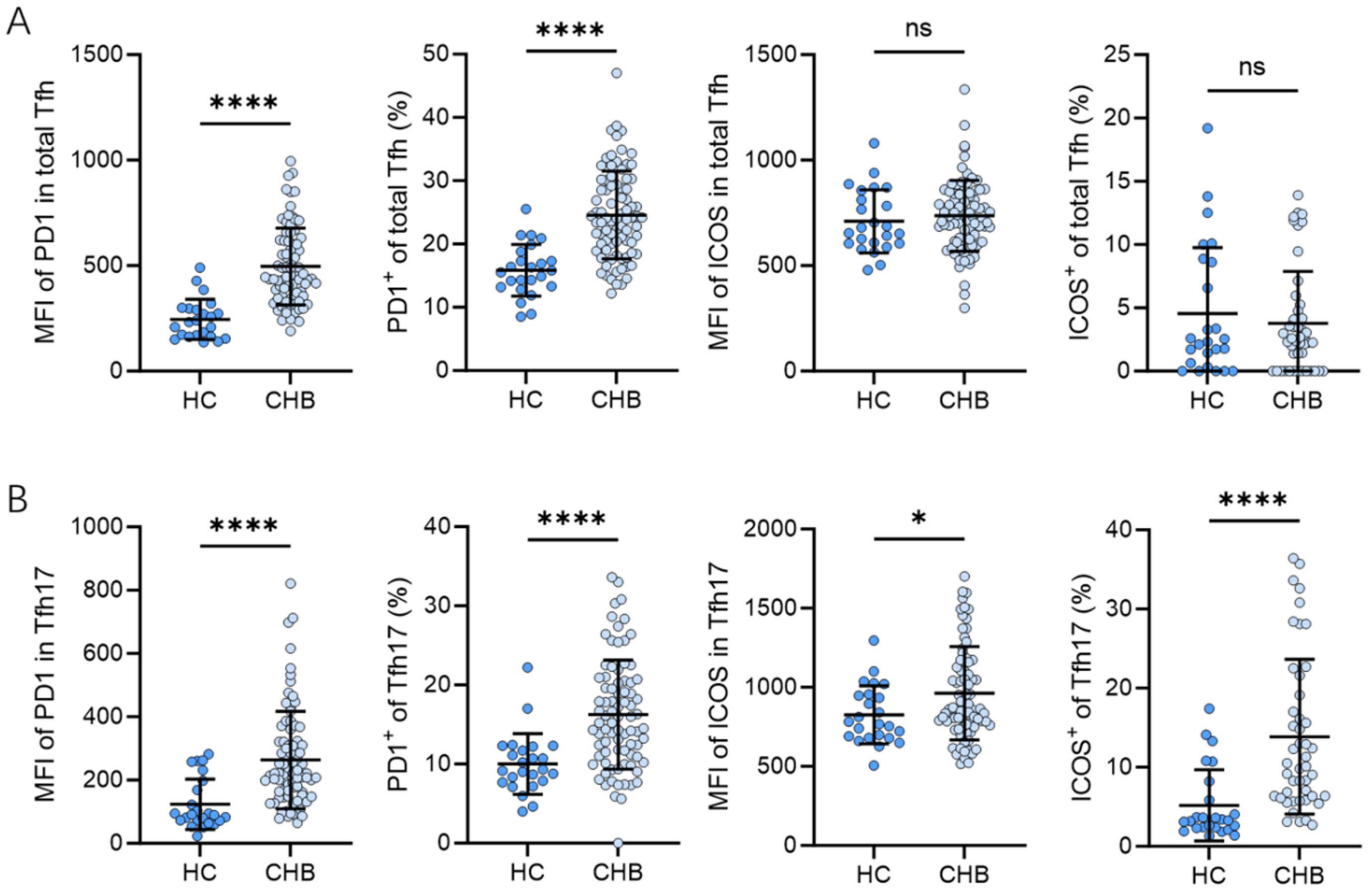
PD-1, ICOS expression in Tfh cell subsets of CHB patients (*n* = 68) and the HC group (*n* = 20); Mann–Whitney *U* test, bars represent medians with IQR. PD-1, ICOS expression in Tfh cells **(A)** and Tfh17 **(B)** of CHB patients and the HC group. MFI, mean fluorescence intensity; Tfh, follicular T helper cell; HBV, hepatitis B virus; CHB, chronic hepatitis B; HC, healthy control; PD-1, programmed cell death-1; ICOS, inducible T-cell costimulator. ^*^p < 0.05, ^**^p < 0.01, ^***^p < 0.001, and ^****^p < 0.0001.

Given the crucial roles of PD-1 and ICOS in cTfh differentiation and function, we analyzed their expression on cTfh (Figure 3A) and cTfh17 cells (Figure 3B). The mean fluorescence intensity (MFI) of PD-1 was significantly higher on cTfh cells and cTfh17 cells in CHB patients than in the HC group (cTfh: 233 vs. 454, *p* < 0.0001; cTfh17: 90.5 vs. 221, *p* < 0.0001; medians). Furthermore, the frequencies of PD-1^+^ cTfh cells and PD-1^+^ cTfh17 cells were significantly increased in CHB patients (cTfh: 15.65% vs. 24.00%, *p* < 0.0001; cTfh17: 9.23% vs. 15.20%, *p* < 0.0001; medians). ICOS MFI and frequencies were significantly higher on cTfh17 cells in CHB patients (MFI: 775.5 vs. 865.0, *p* = 0.0385; frequencies: 3.29% vs. 10.30%, *p* < 0.001; medians) but not on total cTfh cells (MFI: 710.1 vs. 736.3, *p* = 0.4908; frequencies: 2.32% vs. 2.49%, *p* = 0.8913; medians) compared to HCs.

### Circulating Tfh17 cell frequency correlates with HBsAg-specific humoral immunity

**Figure 4:**
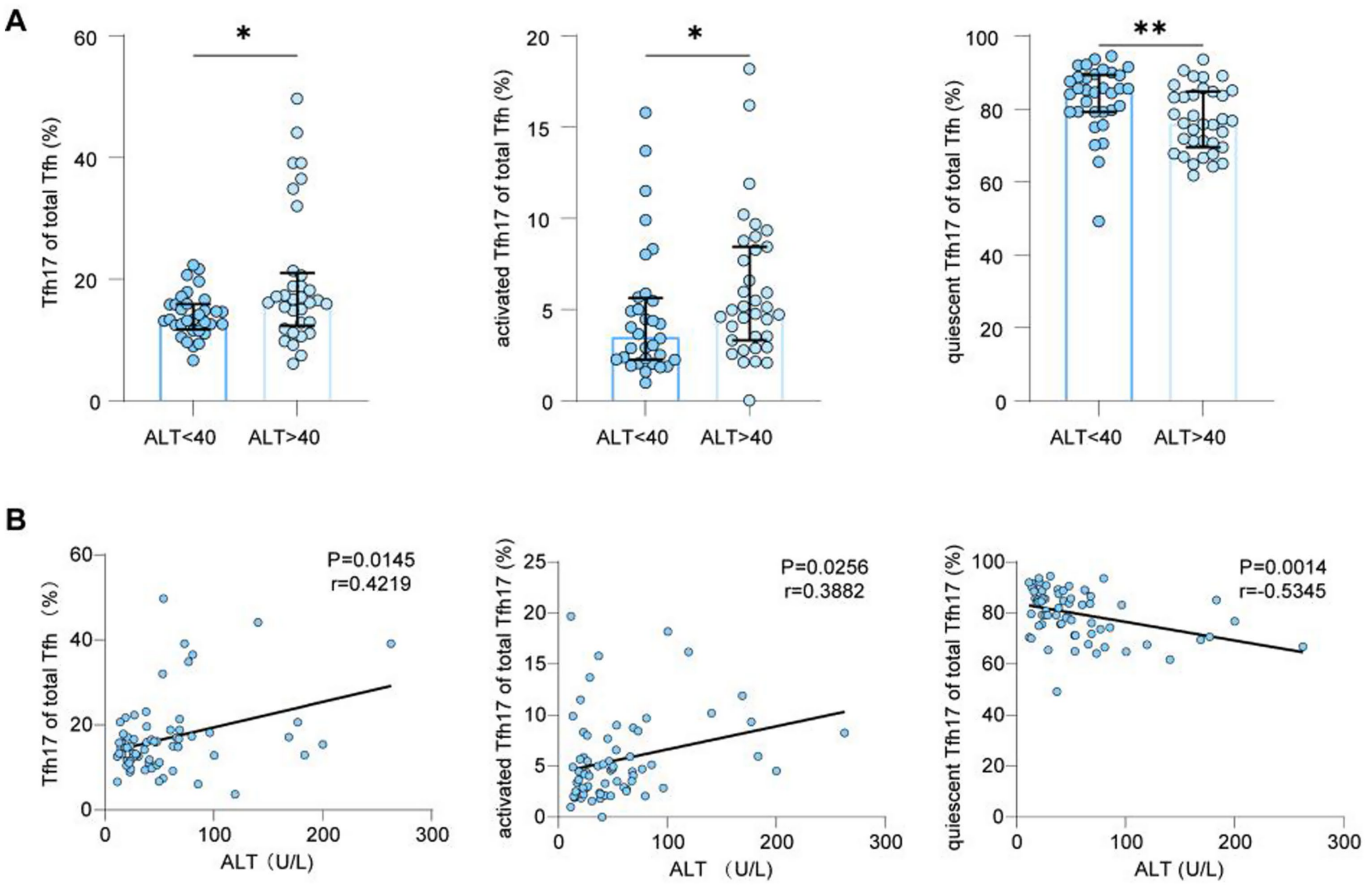
The analysis of Tfh17 frequencies and ALT. **(A)** Frequencies of Tfh cell subsets in patients with CHB at different ALT levels (ALT <40: *n* = 33; ALT >40: *n* = 35); Mann–Whitney *U* test, bars represent medians with IQR. **(B)** Correlation analysis of Tfh17 cells and their subsets with ALT in CHB patients (*n* = 68); Spearman correlation. Tfh, follicular T helper cell; CHB, chronic hepatitis B; ALT, alanine aminotransferase. ^*^p < 0.05, ^**^p < 0.01, ^***^p < 0.001, and ^****^p < 0.0001.

To investigate the role of cTfh17 in CHB, we analyzed correlations between cTfh17 frequencies and clinical indicators. Frequencies of total cTfh17 and activated cTfh17 cells were higher in CHB patients with severe liver inflammation (ALT >40) than in those with less degree of inflammation (ALT ≤40) (cTfh17: 13.30% vs. 16.50%, *p* = 0.0305; activated cTfh17: 3.53% vs. 4.97%, *p* = 0.0363; medians). Conversely, quiescent cTfh17 frequency was lower in patients with ALT >40 U/L (85.20% vs. 76.10%, *p* = 0.0025; medians) (Figure 4A). Correlation analysis confirmed a positive correlation between ALT levels and frequencies of total cTfh17 cells (*p* = 0.0145, *r* = 0.4219) and activated cTfh17 cells (*p* = 0.0256, *r* = 0.3882), but a negative correlation with quiescent cTfh17 cells (*p* = 0.0014, *r* = −0.5345) (Figure 4B). In addition, cTfh1 and cTfh2 frequencies showed no significant correlation with ALT (cTfh1: *p* = 0.2709, *r* = −0.1354; cTfh2: *p* = 0.0767, *r* = −0.2229) (Supplementary Figure S2).

**Figure 5:**
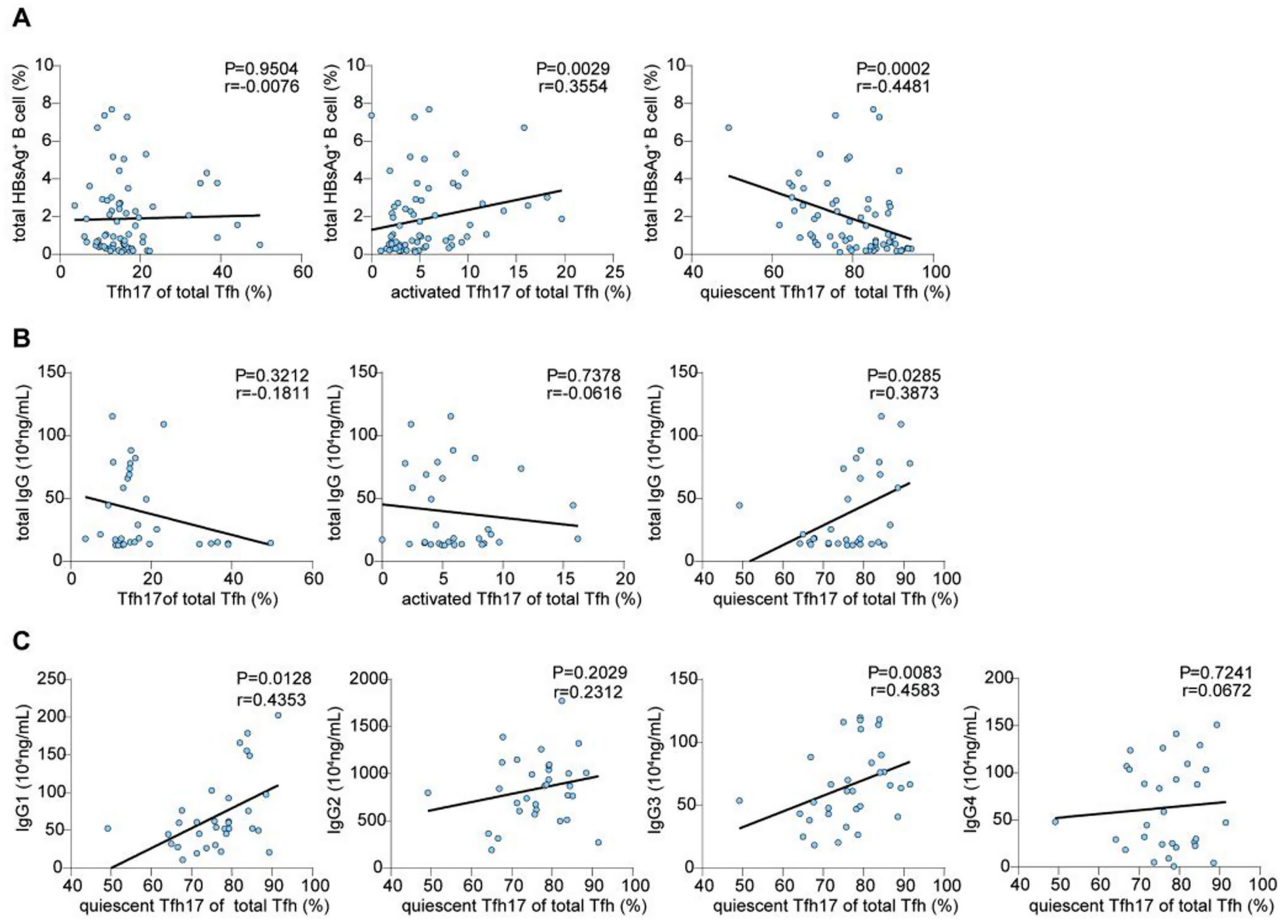
Correlation analysis of Tfh17 cells and their subpopulations in CHB patients; spearman correlation. **(A)** Correlation analysis of Tfh17 cells and their subpopulations with HBsAg^+^ B cells in CHB patients (*n* = 68). **(B)** Correlation analysis of Tfh17 cells and their subpopulations with IgG in CHB patients. **(C)** Correlation analysis of quiescent Tfh17 cells with IgG and their subpopulations in CHB patients. Tfh, follicular T helper cell; CHB, chronic hepatitis B; HBsAg, hepatitis B surface antigen; IgG, immunoglobulin G.

Next, we analyzed HBsAg-specific B-cell frequencies and their relationship with cTfh17 cells. HBsAg-specific B-cell frequencies did not differ significantly between CHB patients and HCs (0.97% vs. 1.32%, *p* = 0.5157) (Supplementary Figure S1). While total cTfh17 frequency showed no correlation with HBsAg-specific B cells (*p* = 0.9504, *r* = −0.0076), activated cTfh17 frequency correlated positively (*p* = 0.0029, *r* = 0.3554) and quiescent cTfh17 frequency correlated negatively (*p* = 0.0002, *r* = −0.4481) with HBsAg-specific B cells (Figure 5A).

Analysis of correlations between cTfh17 frequencies and serum immunoglobulin G (IgG) levels revealed no significant correlations for total cTfh17 (*p* = 0.3212, *r* = −0.1811) or activated cTfh17 (*p* = 0.7378, *r* = −0.0616) (Figure 5B). However, quiescent cTfh17 frequency correlated positively with total IgG (*p* = 0.0285, *r* = 0.3873). Further analysis showed positive correlations between quiescent cTfh17 frequency and IgG1 (*p* = 0.0128, *r* = 0.4353) and IgG3 (*p* = 0.0083, *r* = 0.4583) levels but no correlations with IgG2 (*p* = 0.2029, *r* = 0.2312) or IgG4 (*p* = 0.7241, *r* = 0.0672) (Figure 5C).

### CHB patients exhibit higher HBsAg-specific cTfh17 cell levels

**Figure 6:**
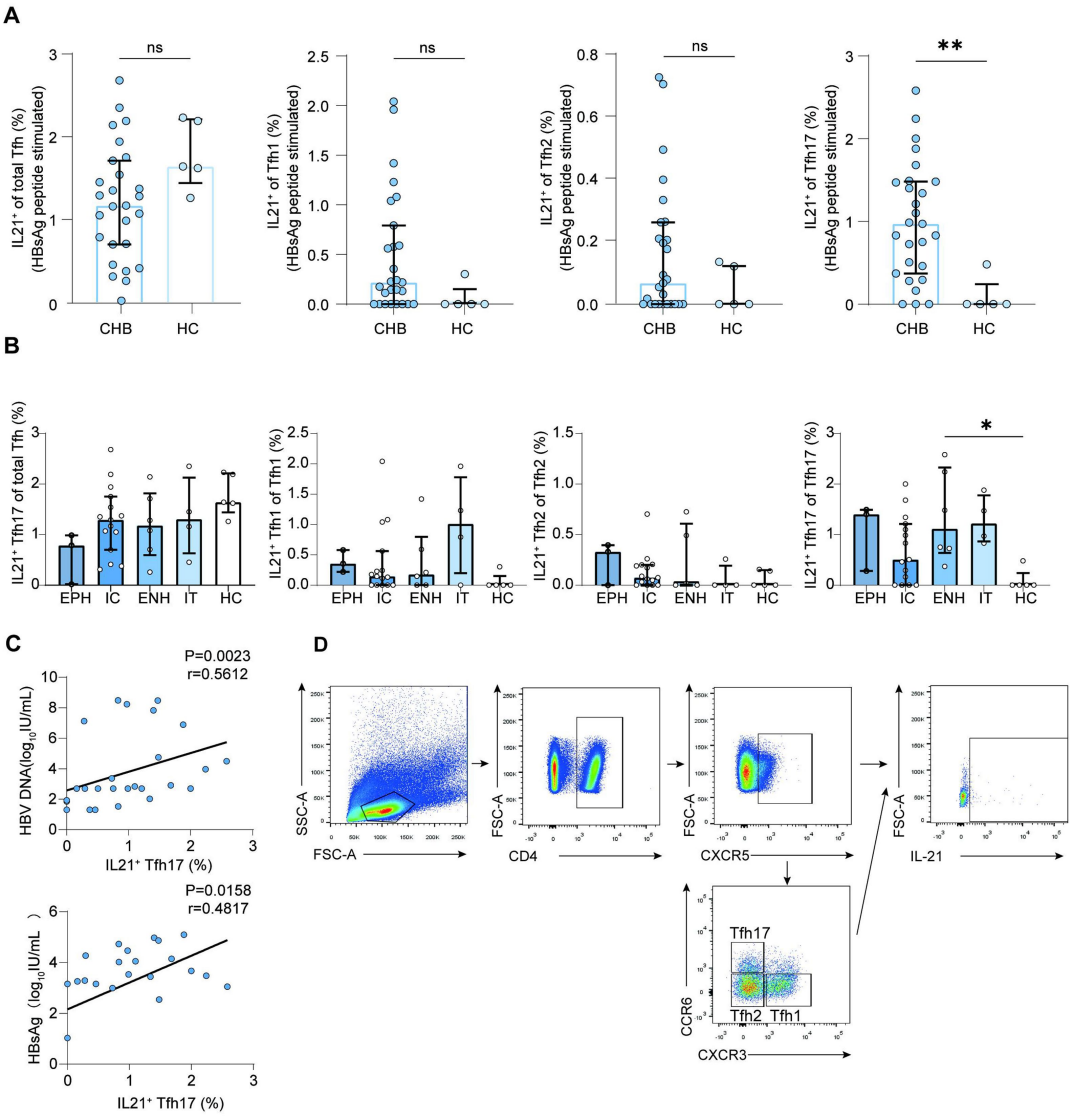
HBsAg-specific IL-21 levels in patients with chronic hepatitis B at different stages of immunity. **(A)** HBsAg-specific IL-21 levels in CHB patients (*n* = 27) and HC group (*n* = 5); Mann–Whitney *U* test, bars represent medians with IQR. **(B)** HBsAg-specific IL-21 levels in CHB patients and HC groups at different immune stages (EPH: *n* = 3, IC: *n* = 14, ENH: *n* = 6, IT: *n* = 4, HC: *n* = 5); Kruskal–Wallis test, bars represent medians with IQR. **(C)** Correlation analysis of HBV DNA, HBsAg, and IL-21 levels (*n* = 24); Spearman correlation. **(D)** Representative gating strategy for IL-21^+^ Tfh cells and Tfh17 cells subset analysis. CHB, chronic hepatitis B; HC, health control; Tfh, follicular T helper cell; HBsAg, hepatitis B surface antigen; IL-21, interleukin-21; IT, immune-tolerant phase; EPH, HBeAg-positive immune-active phase; IC, inactive CHB carrier phase; ENH, HBeAg-negative immune reactivation phase. ^*^p < 0.05, ^**^p < 0.01, ^***^p < 0.001, and ^****^p < 0.0001.

To assess HBsAg-specific cTfh cells, peripheral blood mononuclear cells (PBMCs) from HCs and CHB patients were stimulated *in vitro* with an HBsAg peptide pool. HBsAg-specific cTfh cells, identified as IL-21^+^ cTfh cells, were collected and analyzed after 3 days. While frequencies of HBsAg-specific total cTfh, cTfh1, and cTfh2 cells did not differ between CHB patients and HCs (cTfh: 1.17% vs. 1.63%, *p* = 0.1467; cTfh1: 0.22% vs. 0.00%, *p* = 0.056; cTfh2: 0.06% vs. 0.00%, *p* = 0.2929; medians), HBsAg-specific cTfh17 frequencies were significantly higher in CHB patients (0.97% vs. 0.00%, *p* = 0.0029; medians) (Figure 6A). HBsAg-specific cTfh17 frequency was significantly higher in ENH patients than in HCs (1.16% vs. 0.00%, *p* = 0.0471) (Figure 6B). Furthermore, HBsAg-specific cTfh17 frequency correlated positively with serum HBV DNA (*p* = 0.0023, *r* = 0.5612) and HBsAg levels (*p* = 0.0158, *r* = 0.4817) (Figure 6C), but showed no significant relationship with ALT or AST levels (ALT: *p* = 0.0754, *r* = 0.3479; AST: *p* = 0.3405, *r* = 0.1908) (Supplementary Figure S3). The representative gating strategy for IL-21^+^ Tfh cells and Tfh17 cells subset analysis was showed in (Figure 6D).

## Discussion

Tfh cells play a crucial role in the HBV immune response, facilitating GC B-cell differentiation, plasma cell formation, and specific antibody secretion (Hu et al., 2013; Morita et al., 2011). However, the specific subtypes involved and their mechanisms in HBV immunity remain incompletely understood. In this study, we investigated the frequency, subset characteristics, and clinical correlations of Tfh cells in untreated CHB patients. We found that while total Tfh cell frequency was elevated in CHB patients, Tfh17 frequency was decreased. Importantly, Tfh17 cells exhibited a more activated state in CHB, particularly in the ENH phase, and HBsAg-specific Tfh17 frequency was increased and correlated with HBV DNA and HBsAg levels. Our findings provide new insights into the role of Tfh17 cells in CHB infection, potentially informing immunotherapeutic strategies for functional cure.

Previous studies have reported that altered ratios of Tfh subsets are associated with various autoimmune diseases (Franco et al., 2021; Martín-Nares et al., 2023; Huang et al., 2023). However, research on the diversity of three subpopulations of Tfh cells in HBV is not sufficient yet. Research showed that Tfh17 cells were enriched after HBV-boosting vaccination. And the cTfh17 cells dominated HBsAg-specific cTfh cells in the memory phase on day 28 post-vaccination with a decrease of cTfh1 (Gao et al., 2023). That may mean Tfh17 cells have a function of long-term protection against HBV. Our analysis of the levels and dynamics of Tfh cell subpopulations from CHB patients showed a significant decrease in Tfh17 cells compared with the HC group, while Tfh1 and Tfh2 cells remained unchanged.

Tfh17 cells played an important role in the immune response and seroconversion in CHB patients. The study of (Zhao et al., 2020) found decreased Tfh17 frequency in HBV-associated cirrhosis patients. There was research that found that HBV patients with a higher level of Tfh17 were more likely to undergo HBsAg or HBeAg seroconversion (Vyas et al., 2018). These studies were consistent with our findings. We further demonstrated that CHB patients have lower quiescent Tfh17 but elevated activated Tfh17 cells. Notably, HBsAg-specific Tfh17 cells were increased in CHB patients despite no overall difference in total HBsAg-specific Tfh cells. This may indicate that HBsAg-specific Tfh17 cells play a more important role in the immune response process in CHB patients.

Further analysis of the frequency of Tfh17 cells in different immune phases, the trends of decrease in Tfh and quiescent Tfh cells, and the increase of activated Tfh cells were more significant in the ENH phase of CHB patients, and the patients in the EPH phase also showed a similar trend. In addition, the analysis of ALT levels showed that ALT levels were statistically different from the frequencies of Tfh17, activated Tfh17, and quiescent Tfh17. There was a positive correlation between ALT levels and Tfh17 and a negative correlation with quiescent Tfh17. The frequency of Tfh17 and activated Tfh17 is correlated with ALT levels in the patient with CHB infection, which suggests that the frequency of Tfh17 and activated Tfh17 is correlated with increased liver disease activity. The frequencies of quiescent Tfh17 cells had a negative correlation with HBsAg^+^ B cells, and the frequencies of quiescent Tfh17 cells had a positive correlation with the serum level of IgG. That means the Tfh17 cells may have the function to stimulate B cells to differentiate into memory B cells and produce IgG, which is also consistent with previous reports by (Morita et al., 2011), who found that blood Tfh2 and Tfh17 cells effectively induce immunoglobulin production by naïve B cells through IL-2. PD-1 may play an important role in the influence of quiescent Tfh17 cells on B cells. PD-1 was known as a co-repressor molecule and had the function to promote B-cell exhaustion. A study found that PD-1 blockade partially restored dysfunctional virus-specific B cells in chronic hepatitis B infection (Salimzadeh et al., 2018). The quiescent Tfh17 cells had low expression of PD-1 and may reduce the B-cell exhaustion. Further studies found that PD-1 restricted CXCR3 to promote the enrichment of Tfh cells in GC, and at the same time inhibited TCR signaling to reduce the sensitivity of Tfh cell ligands, reducing the impact of low-affinity or non-specific Tfh on B cells in GC (Shi et al., 2018; Wang et al., 2016). Importantly, the most potent B-cell helper, Tfh17 cells, were significantly reduced in CHB patients, which may link the inefficient antibody response to the pathological B-cell response.

Due to the important role of IL-21 in regulating Tfh differentiation and function, we further determined the frequency of IL-21-positive Tfh cells. The expression of IL-21^+^ Tfh17 was increased in patients with chronic hepatitis B compared to the HC group. IL-21 is a multifunctional cytokine that is involved in the activation of immune cells that contribute to HBeAg seroconversion (Ma et al., 2012). Recent research has found that combined OX40 stimulation and PD-L1 blockade may improve HBV-specific CD4^+^ T cell responses by increasing the secretion of the helper signature cytokines IFN-*γ* and IL-21 (Jacobi et al., 2019). A study that co-cultured blood T cells and B cells found that Tfh2 and Tfh17 cells can induce naive B cells to produce immunoglobulins by IL-21 secretion (Ueno et al., 2015). Furthermore, we found the IL-21 from Tfh17 cells correlated with HBsAg and HBV DNA. In fact, IL-21 plays a key role in HBV infection by activating multiple pathways that support viral suppression and clearance. In CHB patients, studies have shown a significant increase in IL-21^+^ CD4^+^ T cells, and IL-21 produced by CD4^+^ T cells enhances the interferon-gamma (IFN-γ) and perforin expression of CD8^+^ T cells in CHB patients. Meanwhile, IL-21 facilitates B-cell antibody secretion, thereby promoting the clearance and transformation of specific antigens during HBV infection. The relationship between IL-21 and HBV DNA, HBsAg reflects the activation of Tfh17 cells.

We also found increased expression of PD-1 and ICOS on Tfh17 in patients with chronic hepatitis B compared to HC. When functioning as a co-inhibitory receptor engaged by its ligands on antigen-presenting cells, PD-1 can dampen TCR signaling and thereby reduce the sensitivity of Tfh cells to its ligands (Yokosuka et al., 2012; Zinselmeyer et al., 2013). ICOS is an essential marker for Tfh generation and GC formation. It has been reported that ICOS-deficient mice showed significantly reduced GC responses and Tfh cells (Bossaller et al., 2006). The signal of ICOS has a role to upregulate Bcl-6 during dendritic cell (DC) priming, and the expression of Bcl-6 or Blimp-1 decides whether the T cells differentiate to Tfh cells (Choi et al., 2011; Qi et al., 2016). The upregulation of Bcl-6 can further increase PD-1 expression (Poholek et al., 2010). Also, ICOS induced rapid externalization of CD40L from intracellular stores, and GC B cells could upregulate ICOS ligand (ICOSL) under the influence of CD40L, which maintains the contact between GC Tfh cells and B cells (Liu et al., 2015). The upregulated PD-1 and ICOS indicated that the Tfh17 upregulated activity increased, although its percentage decreased.

T follicular helper (Tfh) cells play important roles in hepatocellular carcinoma (HCC) (Zhao et al., 2024; Jia et al., 2022). In HCC, Tfh cells highly express ICOS, which promotes their differentiation and triggers the formation of tertiary lymphoid structures (TLS) (Panneton et al., 2023; Chaurio et al., 2022). Meanwhile, Tfh cells facilitate the differentiation of B cells into plasma cells through CD40 signaling (Luo et al., 2018). These findings are consistent with the function of circulating Tfh cells observed in patients with chronic hepatitis B (CHB). Furthermore, studies have identified abundant infiltration of Tfh cells in HCC tissues, which is associated with lower survival rates. Elevated levels of Tfh-related factors have also been observed in patients with liver fibrosis (Xu et al., 2023; Nalkurthi et al., 2022). In HBV-related HCC, studies have shown increased ICOS expression in circulating Tfh cells and a reduction in the Tfh17 subset (Duan et al., 2015), which aligns with our results. This suggests that an increase in Tfh cells is often associated with more severe liver inflammation, potentially due to their role in promoting humoral immune responses. Some researchers have identified specific cell subsets in CHB and HBV-associated HCC, such as pro-inflammatory (Pro-infla) CD14^+^ monocytes, Pro-infla CD16^+^ monocytes, and IFNG^+^ CX3CR1^−^ NK cells (Jiang et al., 2024; Yin et al., 2023). However, research on Tfh cell subsets remains insufficient and warrants further investigation.

We recognized that our study has some limitations, due to the lack of liver tissue samples; our study was limited to the analysis of circulating Tfh cell responses, but lacked intrahepatic Tfh cells identified in the liver. The subsets of HBsAg-specific Tfh17 may have different effects on the immune response to HBV. It is meaningful to further analyze the subsets of HBsAg-specific Tfh17 cells and their characterizations. PEG-IFN-*α*-2b treatment is an important treatment method for HBV, but the variations in immune status and immune cells of CHB patients after PEG-IFN-α-2b treatment are not clear enough now. Therefore, there is a need to further investigate the exact role of HBV-specific Tfh cell subsets and other immunoreactive cells in the pathogenesis of CHB in patients treated with NAs or in combination with PEG-IFN-α-2b using a larger population. T follicular regulatory cells were an important part of T cells. They had similar cell surface markers to Tfh cells but different functions. Including them in the Tfh cell subgroup may skew the results, and we will consider this in future studies. Also, the unbalanced cohort size in this study constitutes a methodological limitation that may compromise the statistical power and generalizability of the findings.

In summary, the frequency of Tfh17 was slightly decreased, whereas the activation was significantly increased in patients with chronic HBV infection. However, studies on the longitudinal changes in Tfh17 during anti-HBV treatment for CHB are needed. Furthermore, the mechanism by which Tfh17 modulates the immune response to HBV infection still deserves further investigation. Understanding the alterations in Tfh17 can help us to better understand the pathogenesis of CHB and design more effective anti-CHB therapies.

## Materials and methods

### Study subjects

**Figure 7:**
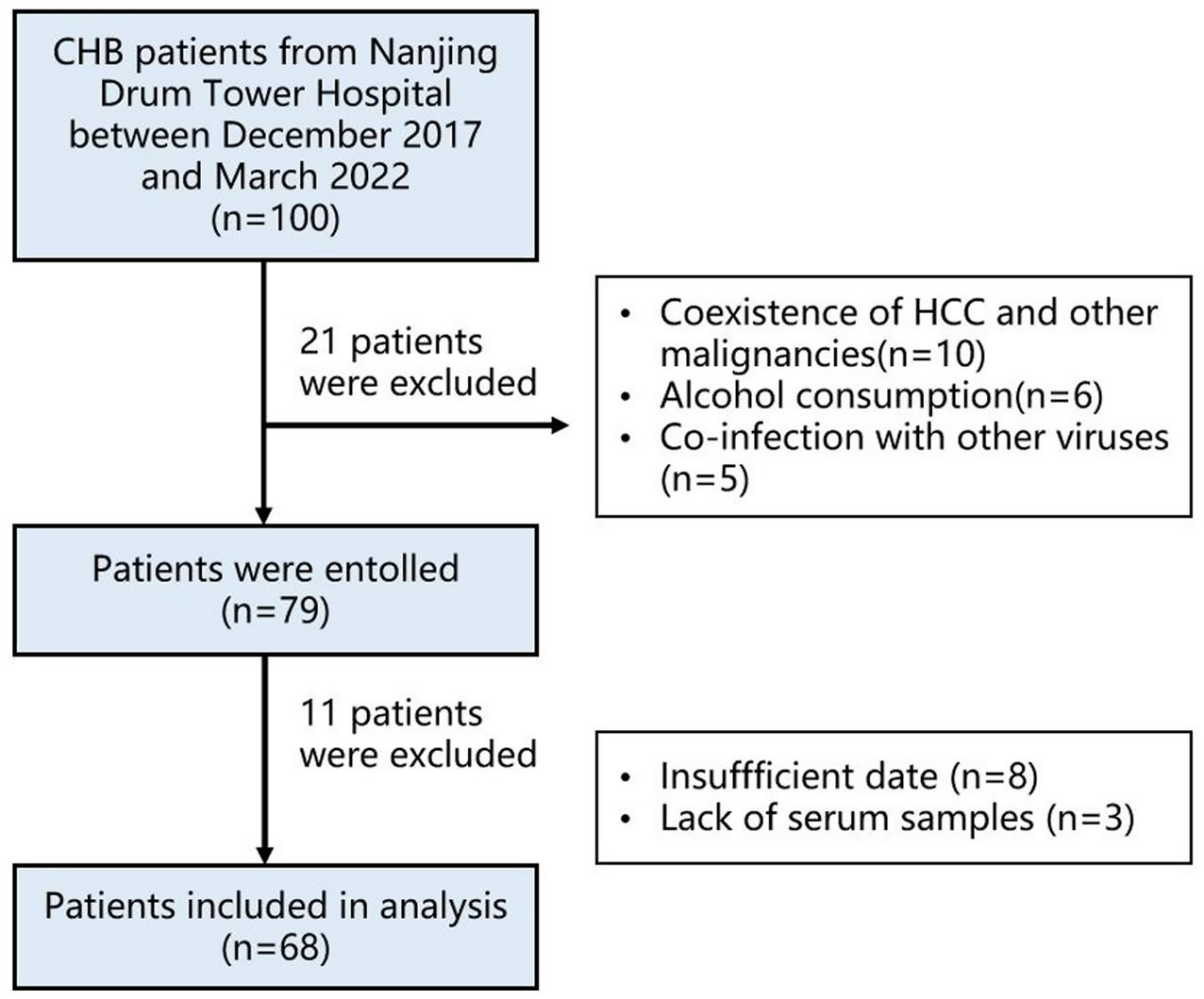
Flow diagram of patient selection. CHB, chronic hepatitis B; HCC, hepatocellular carcinoma.

The study population comprised CHB patients from the outpatient service for hepatitis during December 2017 to March 2022 in Nanjing Drum Tower Hospital who had been positive for HBsAg for more than 6 months. In addition, our cohort included healthy age- and sex-matched adult volunteers who completed immunization with the standard HBV vaccine (three doses) during childhood as controls. Patients who met one of the following criteria were excluded: (1) having received antiviral therapy or immunosuppressive therapy within the past 6 months; (2) co-infection with other viruses, including HCV, HIV, cytomegalovirus, Epstein–Barr virus; (3) evidence of cirrhosis, autoimmune disease, or malignant disease (Figure 7). In accordance with the Declaration of Helsinki, this study was approved by the Institutional Review Committee of Nanjing Drum Tower Hospital. In this research, each subject signed the written informed consent form. According to the AASLD guidelines for treatment of chronic hepatitis B, the patients are divided into four groups: (1) Immune-tolerant phase (IT); (2) HBeAg-positive immune-active phase (EPH); (3) Inactive CHB carrier phase (IC); (4) HBeAg-negative immune reactivation phase (ENH) [41].

### Virological and biochemical assessments

HBsAg, anti-HBsAg, HBeAg, anti-HBeAg, and anti-HBcAg levels were measured using a microsomal enzyme immunoassay (Architect System, Abbott, North Chicago, IL, United States). According to the manufacturer’s instructions, serum HBV DNA load was determined using quantitative real-time PCR (Shenyou Biotech, Shanghai, China). The lower limit for detection of HBV DNA is 20 IU/mL. Serum alanine aminotransferase (ALT) and aspartic aminotransferase (AST) levels were measured by Beckman Coulter Biochemical analyzer (Brea, CA, United States), and 40 U/L was the upper limit of normal (ULN).

### PBMC isolation

PBMC were isolated from 10 mL fresh heparinized peripheral blood using STEMCELL Technologies by density gradient centrifugation. Simply, the blood sample is diluted with PBS, and the mixture is added to the lymphatic vessels. After separating the heart at 2,500 rpm at room temperature for 15 min, the blood was divided into the top layer of plasma, an intermediate layer containing white PBMC (also known as the pale coat), and a lower layer containing polymorphonuclear cells. Carefully aspirate the PBMC from the middle layer for further study.

### ELISA for detecting immunoglobulin G

For IgG detection, a commercial Enzyme-Linked Immunosorbent Assay (ELISA) kit (Thermo Fisher Scientific, catalog BMS2091) was used. Plasma was diluted 500,000 times, and IgG-HRP was added for 60 min of incubation. The plate was then washed 5 times, and TMB chromogen solution was added for 30 min of incubation. Added the stop solution and determined the OD450 value. The antibody titer was calculated using the standard curve generated from a standard with a known titer.

### HBsAg peptide stimulation

The HBsAg peptide pool consists of 47 15-MER synthetic peptides with overlapping 15-amino-acid (aa) residues, covering the entire sequence of genotype B or genotype C HBV surface protein, and was synthesized by Beijing Siguang Biotechnology LLC (Shanghai, China). To detect the frequency and number of Tfh cells secreted by HBsAg-specific IL-21, PBMCs were resuscitated in RPMI 1,640 complete medium with 10 μg/mL HBsAg peptide pools for 3 days. An equimolar dose of dimethyl-sulfoxide (DMSO) was used as a negative control and PMA/ionomycin as a positive control. Brefeldin A was added during the last 6 h of culture to enhance the detection of intracellular cytokines.

### Flow cytometry analysis

Peripheral blood cells were stained with CD4 (RM4-5, 1:50), CD19 (eBio1D3, 1:50), IgD (IA6-2, 1:50), CD27 (M-T271, 1:50), CD45 (HI30, 1:50), CXCR5 (RF8B2, 1:50), CCR6 (11A9, 1:50), CTLA4 (UC10-4B9, 1:50), ICOS (DX29, 1:50), PD-1 (NAT105, 1:50), IL-21 (3A3N2, 1:50), and CXCR3 (1C6/CXCR3, 1:50). The antibodies were purchased from BD Bioscience, NJ, United States. The appropriate type of control was parallel to the saturation concentration. Fluorescence-activated cell sorting (FACS) analysis was performed on BD FACS Aria II using FACSDiva software (all from BD Biosciences, San Jose, CA, United States).

### Statistical analysis

Statistical Package for the Social Sciences (SPSS) software version 22.0 (IBM, Armonk, NY, United States) was used to analyze all data. The Mann–Whitney *U* test was used for comparison between groups. The variance between groups was compared by analysis of variance (ANOVA) or the Kruskal–Wallis *H* test. The Spearman rank correlation test was used to evaluate the correlation between variables. A *p*-value <0.05 was considered statistically significant.

## Data Availability

The original contributions presented in the study are included in the article/Supplementary material, further inquiries can be directed to the corresponding authors.
