# Supplementary material for: Activated circulating T follicular helper 17 cells positively correlated with anti-HBV humoral immunity in chronic hepatitis B patients
Source: Front Microbiol. 2026 Jan 13;16:1708034. doi: 10.3389/fmicb.2025.1708034 (PMC12835811; doi:10.3389/fmicb.2025.1708034)

**Figure S1. The HBsAg+ B cell frequencies in patients with CHB (n=68) and in the HC (n=20) group.** CHB, chronic hepatitis B; HC, healthy control; HBsAg, hepatitis B surface antigen. Figure S1: Mann-Whitney U test, bars represented medians with IQR.

**
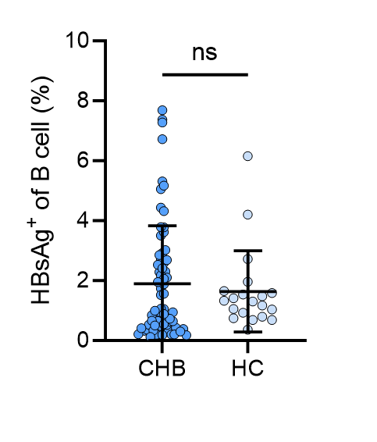
**

**Figure S2. The relationship between ALT and the frequency of Tfh1, Tfh2 cells (n=68).** ALT, alanine aminotransferase. Figure S2: Spearman correlation.


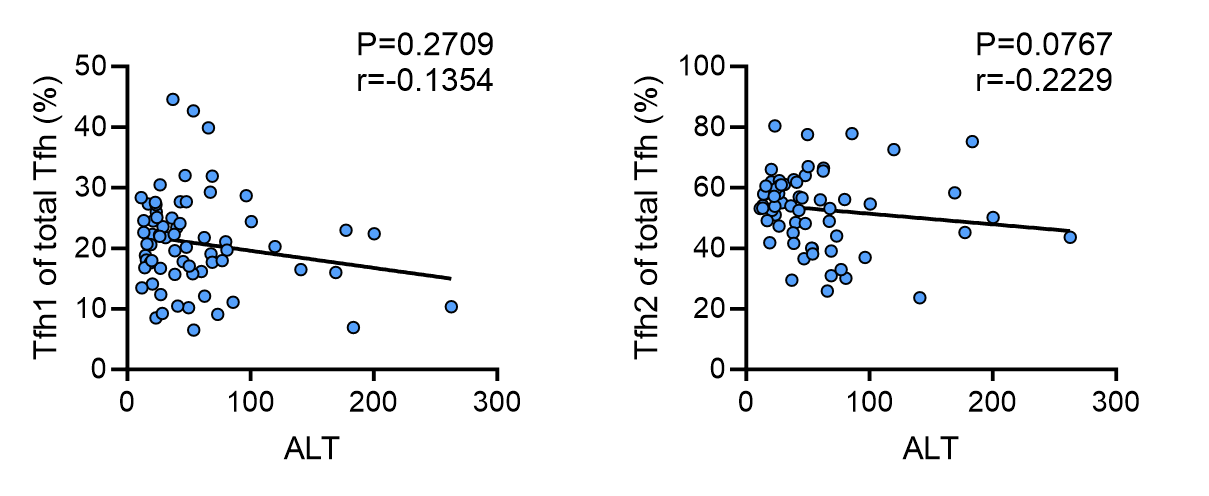


**Figure S3. The relationship between IL-21+ Tfh17 cells and ALT, AST (n=27).** ALT, alanine aminotransferase; AST, aspartate aminotransferase. Figure S3: Spearman correlation.


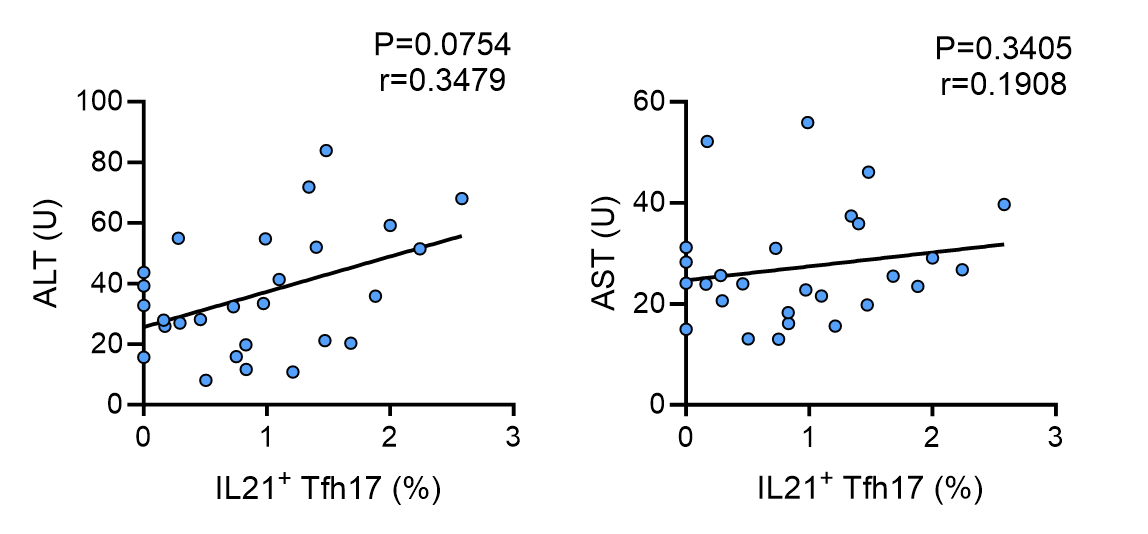


**Figure S4. The counts of Tfh17, activated Tfh17 and quiescent Tfh17 cells (CHB: n=68, HC: n=20).** Tfh, follicular T helper cell. Figure S4: Mann-Whitney U test, bars represented medians with IQR.


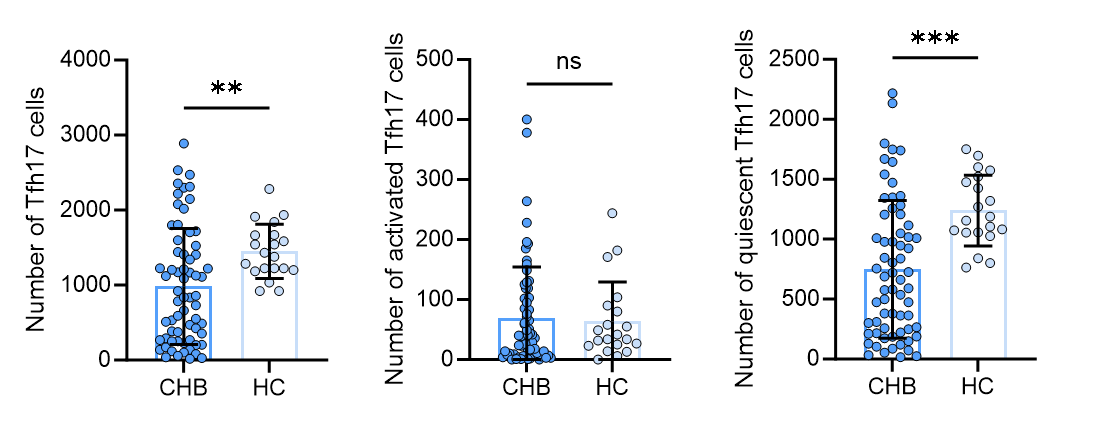


**Figure S5. Expression of ICOS and PD-1 in CHB and HC.**


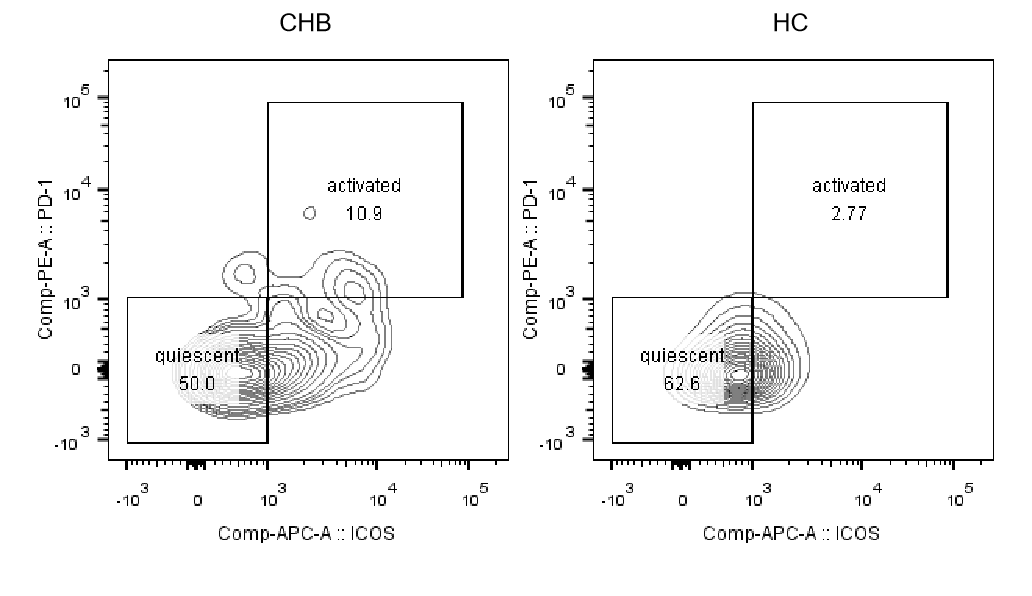

Supplement: Supplementary file 1 [file Supplementary_file_1.docx]
